# Isolation of maltol derivatives from *Stellera chamaejasme* and the anti-atopic properties of maltol on skin lesions in DNCB-stimulated mice†

**DOI:** 10.1039/c8ra09743g

**Published:** 2019-01-15

**Authors:** Beom-Geun Jo, No-June Park, Su-Nam Kim, Jonghwan Jegal, Sangho Choi, Sang Woo Lee, Li Wan Yi, Seoung Rak Lee, Ki Hyun Kim, Min Hye Yang

**Affiliations:** College of Pharmacy, Pusan National University Busan 46241 South Korea mhyang@pusan.ac.kr +82-51-510-2811 +82-51-513-6754; Natural Products Research Institute, Korea Institute of Science and Technology Gangneung 25451 South Korea; International Biological Material Research Center, Korea Research Institute of Bioscience and Biotechnology Daejeon 34141 South Korea; Institute of Medicinal Plants, Yunnan Academy of Agricultural Sciences Yunnan 650205 China; Natural Product Research Laboratory, School of Pharmacy, Sungkyunkwan University Suwon 16419 South Korea khkim83@skku.edu +82-31-290-7730 +82-31-290-7700

## Abstract

The aim of this study was to isolate maltol derivatives from *S. chamaejasme* and to investigate the anti-atopic dermatitis (anti-AD) effect of maltol in a 2,4-dinitrochlorobenzene (DNCB)-sensitized mouse model of AD. A novel compound, maltol 3-*O*-(4′-*O-cis-p*-coumaroyl)-*β*-d-glucoside (named isosoyamaloside I), and two known maltol derivatives (maltol and soyamaloside I) were isolated from *S. chamaejasme* using chromatographic methods. Dermal application of maltol to DNCB-sensitized AD mice reduced erythema, pruritus, and lichenification scores. Histopathological examinations revealed significant decline in mast cell infiltration in maltol-treated AD mice. In addition, maltol accelerated skin barrier recovery by reducing TEWL and skin pH and increasing skin hydration. Maltol was also found to suppress atopy-induced IL-4 and IgE elevations in serum, which are known to be essential for the development of atopy. The results of this study show that maltol is a potential therapeutic candidate for the treatment of AD-related skin diseases.

## Abstract 2

The aim of this study was to isolate maltol derivatives from *S. chamaejasme* and to investigate the anti-atopic dermatitis (anti-AD) effect of maltol in a 2,4-dinitrochlorobenzene (DNCB)-sensitized mouse model of AD.

## Graphical abstract

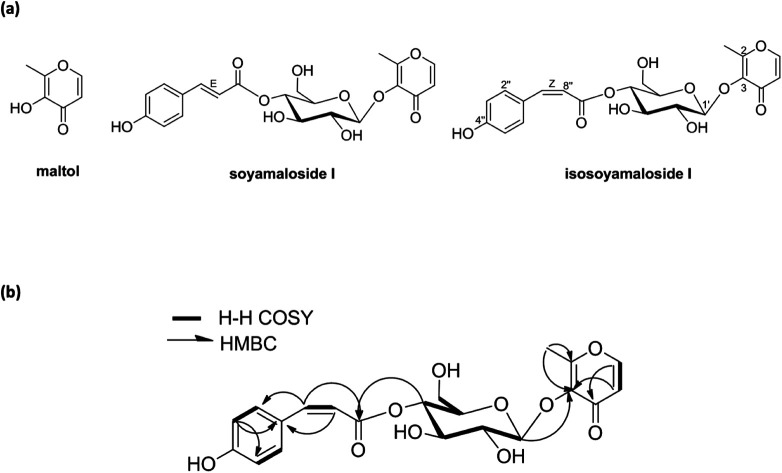

## 1 Introduction

Atopic dermatitis (AD) is a relapsing chronic inflammatory skin disease that exhibits increasing prevalence in childhood.^1^ AD is characterized by episodes of extreme pruritus and features of eczema in localized lesions.^2,3^ Atopy-specific helper T cells (T_H_2-like T cells) play an important role in the initiation of allergic immune response.^4,5^ Preferential accumulation of T_H_2 cells in acute skin lesions of AD results in significant increases in the numbers of cells expressing IL-4, IL-5, and IL-13.^3,4^ In particular, expression of transgenic IL-4 has been reported to decrease the expressions of multiple genes associated with innate defense, and to trigger an allergic inflammation that resembles AD in human skin.^6,7^ Topical applications of the immunomodulators tacrolimus and pimecrolimus inhibit the expressions of the inflammatory cytokine IL-4, and today the use of nonsteroidal topical agents is viewed to have considerable potential for the treatment of inflammatory skin conditions and atopic diseases.^8,9^ In this reason, the down-regulation of IL-4 overexpression in AD is considered an important pharmacological target.^10,11^

*Stellera chamaejasme* L. (Thymelaeaceae) is a multi-stemmed perennial weed mainly distributed in northern and southwestern China.^12^ The roots of *S. chamaejasme*, also known as ‘*Langdu*’, are used in traditional medicines in many Asian countries, including China, Nepal, India and Japan, as a remedy for stubborn skin ulcers, psoriasis, chronic tracheitis, and tuberculosis.^13,14^ Extracts of *S. chamaejasme* have been reported to exhibit anti-inflammatory, analgesic, and wound healing activities.^13,15^ and phytochemical investigations of the roots of *S. chamaejasme* have identified flavonoids, diterpenes, and coumarins as main constituents.^16,17^ However, no detailed studies have been conducted to determine whether on maltol or on the maltol glycosides, which are found in the aerial parts of *S. chamaejasme*, have anti-AD properties. During our continuing search for anti-inflammatory constituents in plants, we identified maltol and a new compound isosoyamaloside I in an ethanoic extract of the aerial parts of *S. chamaejasme* and found they acted as strong IL-4 inhibitors. Additional study was undertaken to investigate the anti-atopic effects of maltol, which was the most active IL-4 inhibitor found, in a 2,4-dinitrochlorobenzene (DNCB)-sensitized murine model of AD.

## 2 Experimental section

### 2.1. General methods for compound isolation

^1^H and ^13^C NMR, COSY, HMQC, HMBC, and NOESY spectral data were obtained using an Agilent Superconducting FT-NMR 400–500 MHz Spectrometer System. HR-ESI mass spectra were recorded on an Agilent Technologies, 6530 Accurate-Mass Q-TOF LC/MS. The HPLC system (Shimadzu, Japan) used was equipped with a pump (Model LC-20AT), an UV detector (Model SPD-20A at 254 nm), and a data station (Model CBM-20A). Column chromatography was performed using silica gel (230–400 mesh, Merck, Germany) and Sephadex LH-20 gel (25–100 μM mesh, Pharmacia, Sweden).

### 2.2. Isolation of maltol and maltol glycosides from *Stellera chamaejasme*

The aerial parts of *Stellera chamaejasme* L. were collected in Yunnan province, Diqing prefecture, Xiang Ge Li Ra, China and identified by Dr Sang Woo Lee (Korea Research Institute of Bioscience and Biotechnology). The name *Stellera chamaejasme* L. was verified using the World Checklist of Selected Plant Families (http://www.theplantlist.org/tpl1.1/record/tro-32000469) and is included in the Plant List (http://www.theplantlist.org). A voucher specimen (PNU-0023) was deposited at the Medicinal Herb Garden, Pusan National University.

Dried aerial parts of *S. chamaejasme* (6.11 kg) were extracted with 95% EtOH and evaporated under reduced pressure to yield *S. chamaejasme* EtOH extract (SCAE) (569.8 g). SCAE was suspended in distilled water and sequentially partitioned using *n*-hexane, ethyl acetate, and *n*-butanol. The ethyl acetate fraction (20.8 g) was then subjected to silica gel column chromatography using a CH_2_Cl_2_–MeOH gradient (20 : 1 → 100% MeOH) to yield 13 fractions (SCE1∼SCE13). Fraction SCE3 (868.4 mg) was suspended in MeOH and filtered to obtain maltol (81.5 mg). Fraction SCE7 (229.1 mg) was suspended in MeOH and filtered to yield soyamaloside I (82.3 mg). The resulting filtrate of SCE7 was subjected to Sephadex LH-20 (MeOH) column chromatography and afforded isosoyamaloside I (33.4 mg).

### 2.3. Cell culture

RBL-2H3 cells (a rat basophilic leukemia cell line) were purchased from the American Type Culture Collection (ATCC, #CRL-2256, Bethesda, MD). Cells were cultured in minimum essential medium (MEM) supplemented with Eagle's salt containing 10% fetal bovine serum (FBS), 2 mM l-glutamine, and antibiotics (100 U mL^−1^ penicillin and 100 μg mL^−1^ streptomycin) at 37 °C in a humidified 5% CO_2_ atmosphere.

### 2.4. IL-4 gene expression in RBL-2H3 cells

RBL-2H3 cells were treated with DMSO or maltol derivatives (10 μM) for 30 min before inflammation was induced with PMA/ionomycin (PI), which induced a state similar to AD. After 16 h of PI treatment, cells were harvested to measure IL-4 mRNA levels by quantitative real-time PCR (Q-PCR). Briefly, total RNA was isolated from the RBL-2H3 cells using RNAiso Reagent (TaKaRa, Shiga, Japan), according to the manufacturer's instructions. The following primer sequence was used: IL-4 forward: 5′-ACC TTG CTG TCA CCC TGT TC-3′; IL-4 reverse: 5′-TTG TGA GCG TGG ACTCAT TC-3′; β-actin forward: 5′-TCA TCA CCA TCG GCA ACG-3′, β-actin reverse: 5′-TTC CT GAT GTC CAC GTC GC-3′. Data analyses were performed using 7500 System SDS software version 1.3.1 (Applied Biosystems).

### 2.5. β-Hexosaminidase release activity in RBL-2H3 cells

RBL-2H3 cells were incubated in a 24-well plate (1 × 10^5^ cells per well) at 37 °C for 2 h, and then incubated with anti-DNP IgE (anti-dinitrophenyl-immunoglobulin E) overnight. These IgE-sensitized cells were then washed with Siraganian buffer, treated with DMSO or maltol derivatives (10 μM) for 1 h, and incubated in DNP-BSA (1 μg mL^−1^) for 30 min to stimulate cell degranulation. To measure β-hexosaminidase activities, culture media were centrifuged and the supernatants obtained were mixed with 1 mM poly-*N*-acetyl glucosamine (p-NAG) in 0.1 M sodium citrate buffer and incubated for 1 h at 37 °C. β-Hexosaminidase release to supernatant were determined by measuring differences in absorbance at 405 nm *vs.* non-treated controls.

### 2.6. Animals

SKH-1 hairless female mice (6 weeks old) were purchased from Orient Bio Inc. (Seongnam, Republic of Korea). Animals were maintained for 1 week in an air-conditioned animal room (25 ± 5 °C; 55 ± 5% RH) under a 12 h on/12 h off lighting cycle. Mice were given access to standard laboratory diet and water *ad libitum*. All experimental procedures were performed in accordance with the Guide for the Care and Use of Laboratory Animals published by the US National Institute of Health (NIH Publication no. 85-23, 2011 revision) and were approved by the Institutional Animal Care and Use Committee of KIST (Certification no. KIST-2016-011).

### 2.7. DNCB-induced atopic dermatitis in hairless mice and maltol treatment

To induce AD-like skin lesions, 1% 2,4-dinitrochlorobenzene (DNCB, 100 μL) (Sigma-Aldrich, Seoul) was applied daily onto the dorsal skins of SKH-1 hairless mice for 7 days (experimental days 1 to 7 (ED0-7); sensitization period). After the first challenge, dorsal skins were challenged with 0.1% DNCB (100 μL) three times per week for 2 weeks (until ED21), and during the same 2 week period 0.5% maltol (100 μL in propylene glycol : EtOH = 7 : 3) was applied to dorsal skins twice a day. When DNCB and maltol were administered on same day, maltol was administered 4 h before DNCB. Animals (*n* = 7) were allocated to one of the following four groups: the vehicle control group (CON), members of which were treated with PG/EtOH (without maltol); the DNCB group (DNCB), which were treated with 1% DNCB from ED0-21; the DNCB/maltol group (DNCB–maltol), which were treated with 1% DNCB from ED0-7 and then co-treated with 0.1% DNCB and 0.5% maltol from ED8-21; and the DNCB/Elidel group (DNCB–Elidel), which were treated with 1% DNCB from ED0-7 and then co-treated with 0.1% DNCB and 1% Elidel cream from ED8-21. PG/EtOH (without maltol) was applied to control mice (CON) and DNCB-treated controls (DNCB controls). Animals were sacrificed on ED21 when dorsal skins were removed and subjected to histopathological examination and blood was collected from abdominal aortas for serum IgE and IL-4 analyses.

### 2.8. Histological analysis

Histological analysis was performed using formalin-fixed paraffin sections (4–6 μm). Briefly, dorsal skins of SKH-1 hairless mice were fixed in 10% neutral formalin for 24 h, embedded in paraffin, cut into 2–3 mm slices, sectioned using a microtome, mounted on slides, and dried overnight at 37 °C. Slices were stained with hematoxylin and eosin to evaluate eosinophil infiltration or with toluidine blue to count mast cells number. Histopathological changes were examined under a light microscope (Olympus CX31/BX51, Olympus Optical Co., Tokyo) and photographed (TE-2000U, Nikon Instruments Inc., Melville, USA).

### 2.9. Transepidermal water loss and skin hydration

Transepidermal water loss (TEWL) was assessed using a Tewameter TM210 unit (Courage and Khazaka, Cologne, Germany) and skin hydration and pH were measured using a SKIN-O-MAT (Cosmomed, Ruhr, Germany). Biophysical skin parameters, that is, TEWL, skin hydration, and skin pH were measured weekly under standard conditions (25 ± 5 °C, 55 ± 5% RH).

### 2.10. Determinations of total serum IgE and IL-4 concentrations

Blood was collected from abdominal aortas and centrifuged at 10 000 rpm for 15 min at 4 °C. Serum samples prepared from blood obtained on ED21 and stored at −70 °C until required for IgE and IL-4 determinations. Total serum IgE and IL-4 levels were quantified using an enzyme-linked immunosorbent assay kit (eBioscience, San Diego).

### 2.11. Statistical analysis

The analysis was conducted using one-way analysis of variance (ANOVA). Results are expressed as means ± SDs, and statistical significance was accepted for *p* values of <0.05.

## 3 Results

### 3.1. Isolation of maltol derivatives from *S. chamaejasme* and structural elucidation of isosoyamaloside I

**Fig. 1:**
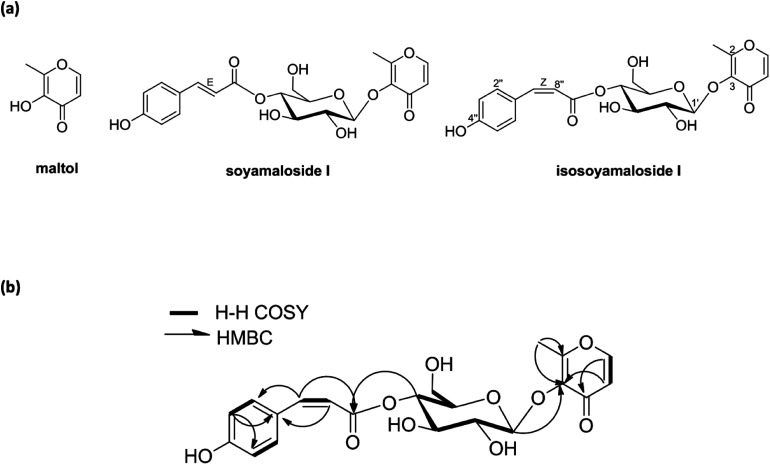
Structures of compounds (maltol, soyamaloside I, and isosoyamaloside I) isolated from *Stellera chamaejasme* (a) and selected ^1^H–^1^H COSY and HMBC correlations of isosoyamaloside I (b).

A new maltol glucoside (isosoyamaloside I) and two known maltol derivatives (maltol and soyamaloside I) were isolated from the 95% EtOH extract of the aerial parts of *S. chamaejasme* (SCAE; Fig. 1a). Maltol and soyamaloside I were identified by comparing ^1^H NMR, ^13^C NMR, and MS data with literature values (Shikishima 2001; Zhang 2015). Isosoyamaloside I was isolated as a white amorphous powder and exhibited negative optical rotation ([*α*]_D_^22^ −53.9; *c* 0.1, MeOH). The molecular formula of isosoyamaloside I was determined to be C_21_H_22_O_10_ by negative-ion HRESIMS (*m*/*z* 433.1173 [M −  H]^−^, calcd for C_21_H_21_O_10_, 433.1213). The IR spectrum of isosoyamaloside I displayed three significant absorption bands of hydroxyl group (3351 cm^−1^), unsaturated ketone (1645 cm^−1^), and aromatic ring (1605, 1513 and 1447 cm^−1^), respectively. Analysis of its ^1^H NMR data (Table 1) suggested the presence of a *cis-p*-coumaroyl moiety [*δ* 5.78 (1H, d, *J* = 12.8 Hz, H-7′′), 6.73 (2H, d, *J* = 8.8 Hz, H-3′′ and H-5′′), 7.67 (2H, d, *J* = 8.8 Hz, H-2′′ and H-6′′)], a *β*-d-glucopyranosyl group at *δ* (1H, d, *J* = 8.0 Hz, H-1′), and a maltol moiety [*δ* 2.45 (3H, s, H-2), 6.43 (1H, d, *J* = 5.6 Hz, H-5), and 7.99 (1H, d, *J* = 5.6 Hz, H-6)]. ^1^H–^1^H COSY, HMQC, HMBC, and NOESY enabled full assignments of ^1^H and ^13^C signals. The HMBC spectrum between the anomeric proton H-1′ (glucose) and C-3 confirmed the existence of a glucopyranosyl group at position of C-3 of maltol. Furthermore, HMBC correlations between glucoside proton H-4′ and C-9′′ confirmed the position of d-glucose in isosoyamaloside I (Fig. 1b). Based on this spectral data, the compound was identified as maltol 3-*O*-(4′-*O-cis-p*-coumaroyl)-*β*-d-glucopyranoside named isosoyamaloside I.

**Table 1:** ^1^H (400 MHz) and ^13^C (100 MHz) NMR spectral data of isosoyamaloside I in DMSO-*d*_6_

Position	Isosoyamaloside I	
	*δ*_H_ (*J* in Hz)	*δ*_C_
2		161.38
3		141.71
4		174.10
5	6.46 (d, 5.6)	116.26
6	8.15 (d, 5.6)	155.79
1′	4.87 (d, 7.8)	103.29
2′	3.32 (m)	74.16
3′	3.45 (m)	73.62
4′	4.69 (dd, 8.0, 8.0)	70.62
5′	3.32 (m)	74.76
6′	3.72 (m)	60.66
1′′		125.37
2′′	7.69 (d, 8.8)	132.77
3′′	6.76 (d, 8.8)	114.88
4′′		158.88
5′′	6.76 (d, 8.8)	114.88
6′′	7.69 (d, 8.8)	132.77
7′′	6.89 (d, 12.8)	143.71
8′′	5.77 (d, 12.8)	115.17
9′′		165.04
2–CH_3_	2.38 (s)	15.24

### 3.2. Maltol inhibited IL-4 expression and mast cell degranulation in RBL-2H3 cells

**Fig. 2:**
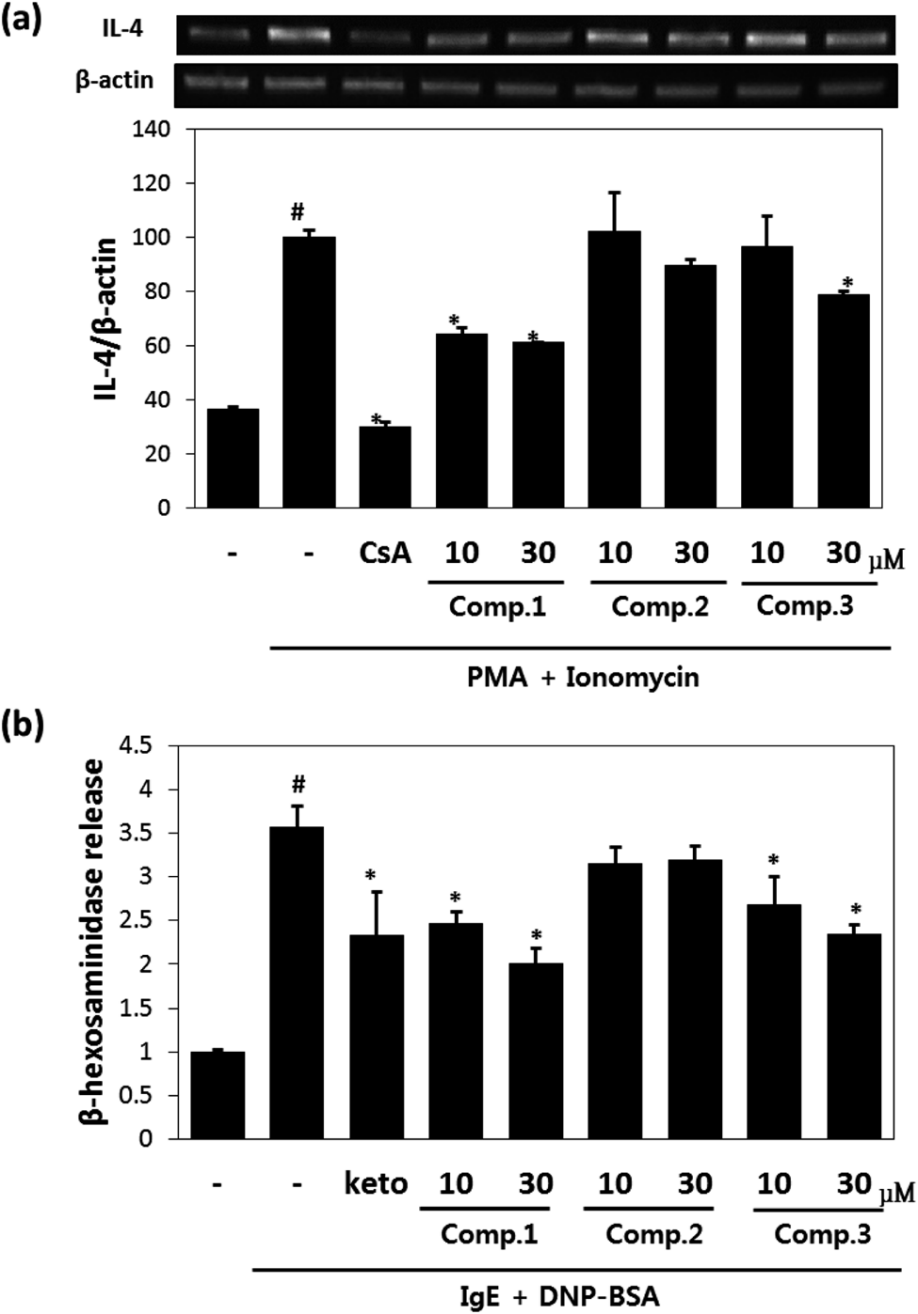
Anti-inflammatory effects of maltol, soyamaloside I, and isosoyamaloside I in RBL-2H3 cells. Effects of maltol, soyamaloside I, and isosoyamaloside I on IL-4 mRNA expression in PI-mediated RBL-2H3 cells (a) and effects of maltol, soyamaloside I, and isosoyamaloside I on β-hexosaminidase release from IgE-mediated RBL-2H3 cells (b). Results are expressed as the means ± SDs of two independent experiments. ^#^*p* < 0.05 *vs.* vehicle control; *p < 0.05 *vs.* PI. CsA: 1 μM cyclosporin A (a); keto: 35 μM ketotifene (b).

Maltol significantly reduced the amounts of IL-4 released by mast cells (Fig. 2a), and inhibited IL-4 release from PI-stimulated RBL-2H3 mast cells by 58% and 67% inhibition at concentrations of 10 μM and 30 μM, respectively. Isosoyamaloside I inhibited IL-4 release (by 40%) at 30 μM, but not at 10 μM. β-Hexosaminidase release is a marker of mast cell degranulation and is a good indicator of the extent of allergic reaction.^18^ The effects of maltol, soyamaloside I, and isosoyamaloside I on β-hexosaminidase release by RBL-2H3 cells were examined, and both maltol and isosoyamaloside I effectively inhibited β-hexosaminidase release from mast cells (Fig. 2b), though maltol had the greater effect.

### 3.3. Effects of maltol on AD-like skin lesions of DNCB-induced atopic mice

**Fig. 3:**
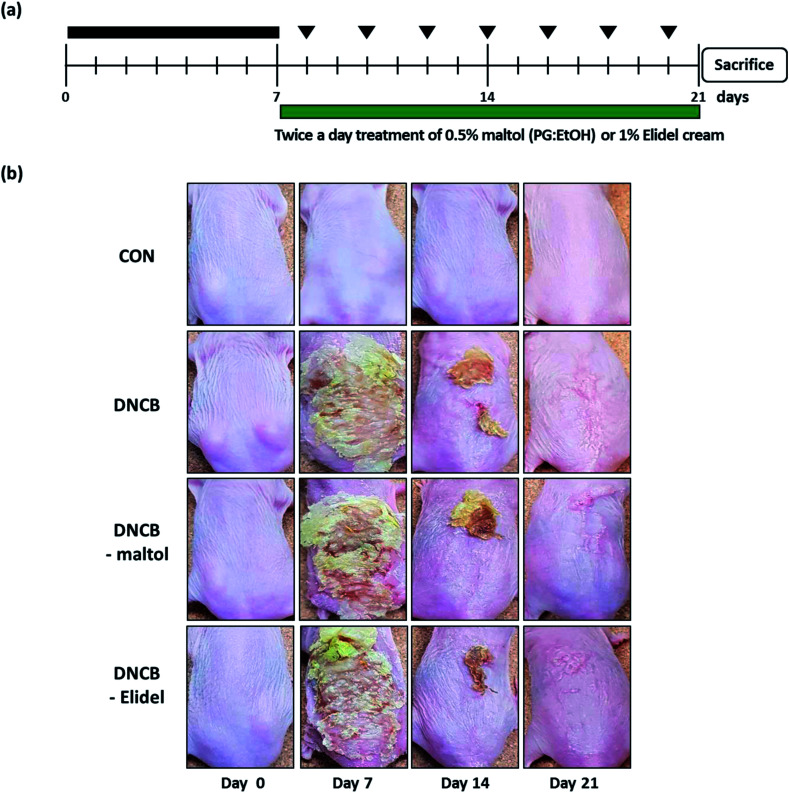
Effects of maltol on pathological changes in the skins of DNCB-sensitized atopic hairless mice. Schematic representation of the experiment (a) and clinical features of AD-like dorsal skin lesions (b). CON: vehicle controls, DNCB: DNCB–controls, DNCB–maltol: DNCB plus 0.5% maltol treated mice, and DNCB–Elidel: DNCB plus 1% Elidel treated mice.

**Fig. 4:**
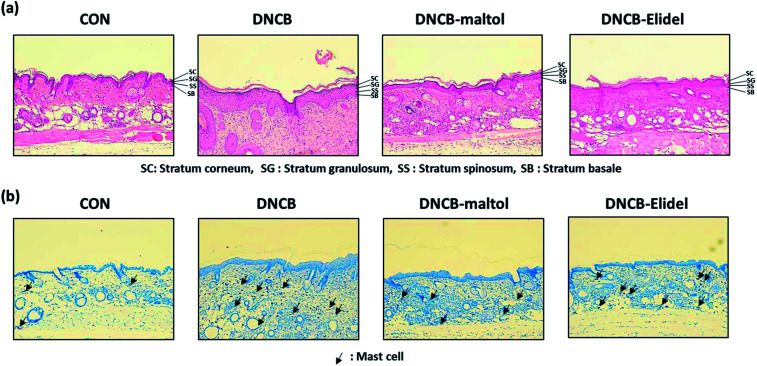
Histopathological effects of maltol in DNCB-sensitized atopic hairless mice. Histopathological features of dorsal skin lesions in 0.5% maltol plus DNCB treated mice as determined by H&E staining (a) and histopathological features of dorsal skin lesions in 0.5% maltol plus DNCB co-treated mice as determined by toluidine blue staining (b). Tissues were excised, fixed in 10% formaldehyde, embedded in paraffin, sectioned and stained with hematoxylin and eosin (H&E) (magnification, 100×) or toluidine blue. Results are presented as means ± SDs (*n* = 7). CON: vehicle controls, DNCB: DNCB–controls, DNCB–maltol: DNCB plus 0.5% maltol treated mice, and DNCB–Elidel: DNCB plus 1% Elidel treated mice. The means ± SEMs of two independent experiments performed in triplicate are shown. ^#^*p* < 0.05 *vs.* vehicle controls; *p < 0.05 *vs.* DNCB treated controls.

Skin lesions were evaluated using dermatitis scores, as summarized in Fig. 3a. On ED20, AD-like skin lesions, such as, erythema, erosion, and dryness, were observed on the dorsal skins of DNCB-treated mice. In contrast, AD-like symptoms were markedly less severe in the DNCB–maltol group than in the DNCB group (Fig. 3b). Dorsal skin sections collected on day 21 were H&E stained to detect cells infiltrating tissues or toluidine blue stained to evaluate mast cell numbers. AD-like symptoms, such as, epithelial and dermal thickening and inflammatory cell infiltration, were observed in the DNCB group. Numbers of inflammatory cells that infiltrated corium tissues and epidermal thickening were significantly lower in the DNCB–maltol group than in the DNCB controls (Fig. 4a), and similar effects were also observed in toluidine blue stained sections of dorsal skin lesions. Mast cell degranulation and infiltration were significantly greater in DNCB controls than in vehicle controls. However, degrees of mast cell infiltration were markedly lower in the DNCB–maltol group than in DNCB controls (Fig. 4b). Application of 1% pimecrolimus cream (Elidel) as a positive control improved AD skin symptoms by decreasing dermal thickening (Fig. 4a) and mast cell infiltration (Fig. 4b) in DNCB-induced atopic animal model.

### 3.4. Effects of maltol on skin barrier function in atopic mice

**Fig. 5:**
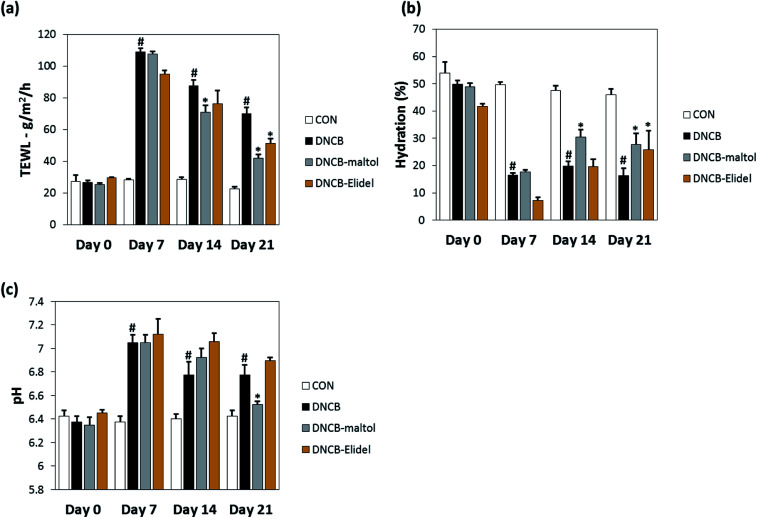
Effects of maltol on skin barrier function in DNCB-sensitized atopic hairless mice. Transepidermal water loss (TEWL) values (a), assessed skin hydration values (b), and skin pH values (c). CON: vehicle controls, DNCB: DNCB–controls, DNCB–maltol: DNCB plus 0.5% maltol treated mice, and DNCB–Elidel: DNCB plus 1% Elidel treated mice. Results are presented as the means ± SDs (*n* = 7) of two independent experiments performed in triplicate. ^#^*p* < 0.05 *vs.* vehicle controls; *p < 0.05 *vs.* DNCB controls.

TEWL and skin hydration values reflect water loss from the body and are commonly as parameters of skin barrier function. TEWL, skin hydration, and skin pH values in stratum corneum on ED0, 7, 14, and 21 are provided in Fig. 5. TEWL and skin pH in the DNCB group were significantly greater than in vehicle controls on ED6. Skin hydration values were lower in the DNCB group than in the vehicle control group on ED21, but TEWL and skin pH values were significantly lower in the DNCB–maltol group than in vehicle controls at this time (by 60% and 75%, respectively) (Fig. 5A and B). Co-treatment with 0.5% maltol was also found to have reduced DNCB-induced skin hydration loss by 29% as compared with DNCB controls (Fig. 5C). On ED21, co-treatment with 1% Elidel, a positive control, reduced the decrease in TEWL observed in the DNCB control by 35% (Fig. 5a) and increased skin hydration by 27% (Fig. 5b).

### 3.5. Effects of maltol on dermal thickness and serum IgE and IL-4 levels in atopic mice

**Fig. 6:**
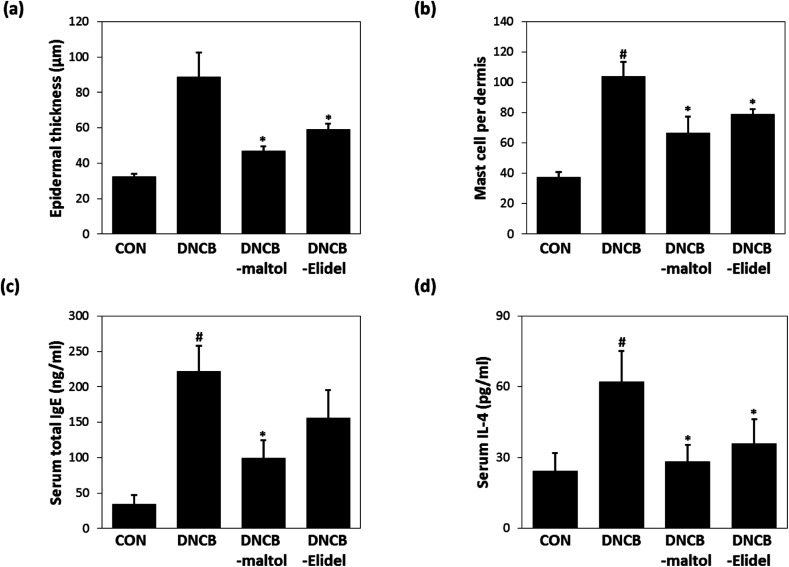
Effects of maltol on dermal thicknesses and on serum IgE and IL-4 concentrations in DNCB-induced atopic hairless mice. Epidermal thicknesses (a), mast cell densities in dermis (b), total serum IgE levels (c), and total serum IL-4 levels (d). CON: vehicle controls, DNCB: DNCB–controls, DNCB–maltol: DNCB plus 0.5% maltol treated mice, and DNCB–Elidel: DNCB plus 1% Elidel treated mice. Results are presented as the means ± standard errors (*n* = 7) of two independent experiments performed in triplicate. ^#^*p* < 0.05 *vs.* vehicle controls; *p < 0.05 *vs.* DNCB controls.

Epidermal thicknesses and mast cell numbers were measured on ED21. As shown in Fig. 6a, dermal skins were significantly thicker in the DNCB-treated group than in vehicle controls. However, maltol significantly reduced these DNCB-induced increases in ear thickness. Maltol significantly inhibited epidermal thickness by 72%. Additionally, mice in the DNCB–maltol group had fewer mast cells in dermis than DNCB controls (Fig. 6b). On ED21, total IgE and IL-4 concentrations were estimated by ELISA. Serum levels of IgE and IL-4 were found to be 8.8- and 3.1-fold higher, respectively, in DNCB controls than in normal controls, but IgE and IL-4 expressions were found to be markedly lower in the DNCB–maltol group than in vehicle controls (Fig. 6c and d). Topical maltol treatment suppressed IgE elevation to 67% of control (Fig. 6c) and IL-4 elevation to 87% of control (Fig. 6d). 1% Elidel co-treatment inhibited DNCB-induced IgE increase by 37% (Fig. 6c) and IL-4 increase by 68% (Fig. 6d).

## 4 Discussion

Maltol (3-hydroxy-2-methyl-4-pyrone) is produced by many plants and contributes to the food flavor.^19,20^ Recently, maltol has attracted research interest because of its antioxidant and anti-fatigue effects in medicinal plants, such as, Korean red ginseng.^21^ Maltol has been reported to have diverse pharmacological actions, such as, neuroprotective and hepatoprotective effects in animals,^22,23^ and maltol and maltol glucoside have been reported to inhibit the overproductions of inflammatory cytokines like TNF-α and IL-1β, and thus, to exhibit anti-inflammatory activities.^23,24^ These research studies shown maltol and maltol derivatives have potential use as therapeutic agents for the treatment of AD. Nevertheless, no reports have been issued on the anti-AD effects of maltol or its derivatives.

During our continuing search for phytochemicals with anti-inflammatory properties, we found that maltol and a novel compound isosoyamaloside I isolated from the aerial parts of *S. chamaejasme* attenuated IL-4 expression and β-hexosaminidase release *in vitro*. We then examined the effects of maltol *in vivo* because it was observed to strongly inhibit mast cell degranulation (RBL-2H3 cells). Topical application of 0.5% maltol was found to suppress AD-like skin symptoms in DNCB-induced hairless mice by reducing leukocyte and mast cell skin infiltration. Interestingly, maltol also significantly decreased skin TEWL and pH values, increased skin hydration, and reduced DNCB-induced increases in epidermal thickness. It has been well established that AD is a disease characterized by altered skin barrier functions and by immune dysregulation.^25^ TEWL and skin hydration values reflect loss of water through skin and are commonly used parameters of skin barrier function.^26^ Furthermore, it has been reported that epidermal cell hyperplasia and decreased skin barrier function are usually followed by an increase in TEWL in murine models of AD.^27^ Our results indicate the anti-AD effect of maltol is closely related to amelioration of skin barrier dysfunction.

Herbal anti-inflammatory agents are often used to prevent and treat skin and allergic diseases.^27^ Plant-derived molecules with anti-AD properties are known to exert their effects by disrupting the activities of inflammatory cytokines and receptors.^27,28^ In the present study, serum IL-4 and IgE levels were elevated in DNCB controls on ED21. However, treatment with maltol from ED7 to ED20 markedly suppressed the serum overexpressions of IL-4 and IgE in atopic mice, which suggests the anti-inflammatory effects of maltol were due at least in part to the suppressions of inflammatory cytokines and IgE synthesis. In particular, IL-4 is a key proinflammatory cytokine in the context of allergic diseases and plays an important role in the induction of IgE synthesis.^29^ Immunosuppressive drugs, tacrolimus, and pimecrolimus selectively target mast cells and inhibit IL-4 release and mast cell degranulation.^5,30^ In contrast to corticosteroids, these topical calcineurin inhibitors were developed as maintenance therapies for AD and do not induce adverse effects, such as, steroid-induced skin atrophy, secondary infections, and acne.^30,31^ Taken together, our findings indicate that the strong IL-4 inhibitory effect of maltol means it has potential use for the treatment of AD.

In conclusion, a new compound, maltol 3-*O*-(4′-*O-cis-p*-coumaroyl)-*β*-d- glucopyranoside (isosoyamaloside I) and two known compounds (maltol and soyamaloside I) were isolated from the aerial parts of *S. chamaejasme*. Maltol most potently inhibited IL-4 levels in RBL-2H3 cells and suppressed the development of atopic dermatitis-like symptoms in our DNCB-induced mouse model of AD. Furthermore, DNCB-induced increases in TEWL and skin pH values were reduced by applying maltol to skin. We believe the observed anti-atopic effect of maltol was probably responsible for the observed enhancements of epidermal barrier functions. Accordingly, we suggest that maltol be considered a developmental candidate for novel anti-inflammatory or immunomodulatory drugs.

## Conflicts of interest

There are no conflicts of interest to declare.

## Supplementary Material

RA-009-C8RA09743G-s001
