# Supplementary material for: Isolation of maltol derivatives from Stellera chamaejasme and the anti-atopic properties of maltol on skin lesions in DNCB-stimulated mice
Source: RSC Adv. 2019 Jan 15;9(4):2125–32. doi: 10.1039/c8ra09743g (PMC9059894; doi:10.1039/c8ra09743g)

Electronic Supplementary Material (ESI) for RSC Advances.
This journal is © The Royal Society of Chemistry 2018

SUPPLEMENTARY DATA

Isolation of maltol derivatives from *Stellera chamaejasme* and the anti-atopic properties of maltol on skin lesions in DNCB-stimulated mice

Beom-Geun Jo,‡[a] No-June Park,‡[b] Su-Nam Kim,[b] Jonghwan Jegal,[a] Sangho Choi,[c] Sang Woo Lee,[c] Li Wan Yi,[d] Seoung Rak Lee,[e] Ki Hyun Kim*[e] and Min Hye Yang*[a]

[a]*College of Pharmacy, Pusan National University, Busan 46241, South Korea. E-mail: mhyang@pusan.ac.kr; Tel: +82-51-510-2811; +82-51-513-6754*

[b]*Natural Products Research Institute, Korea Institute of Science and Technology, Gangneung 25451, South Korea*

[c]*International Biological Material Research Center, Korea Research Institute of Bioscience and Biotechnology, Daejeon 34141, South Korea*

[d]*Institute of Medicinal Plants, Yunnan Academy of Agricultural Sciences, Yunnan 650205, China*

[e]*Natural Product Research Laboratory, School of Pharmacy, Sungkyunkwan University, Suwon, 16419, South Korea. E-mail: khkim83@skku.edu; Tel: +82-31-290-7700; Fax: +82-31-290-7730*

Supporting Information Contents:

S1. The ^{1}H NMR spectrum of compound **3**
S2. The ^{13}C NMR spectrum of compound **3**
S3. The ^{1}H-^{1}H COSY spectrum of compound **3**
S4. The ^{1}H-^{1}H NOESY spectrum of compound **3**
S5. The HMQC spectrum of compound **3**
S6. The HMBC spectrum of compound **3**

S1. The ^{1}H NMR spectrum of compound **3**

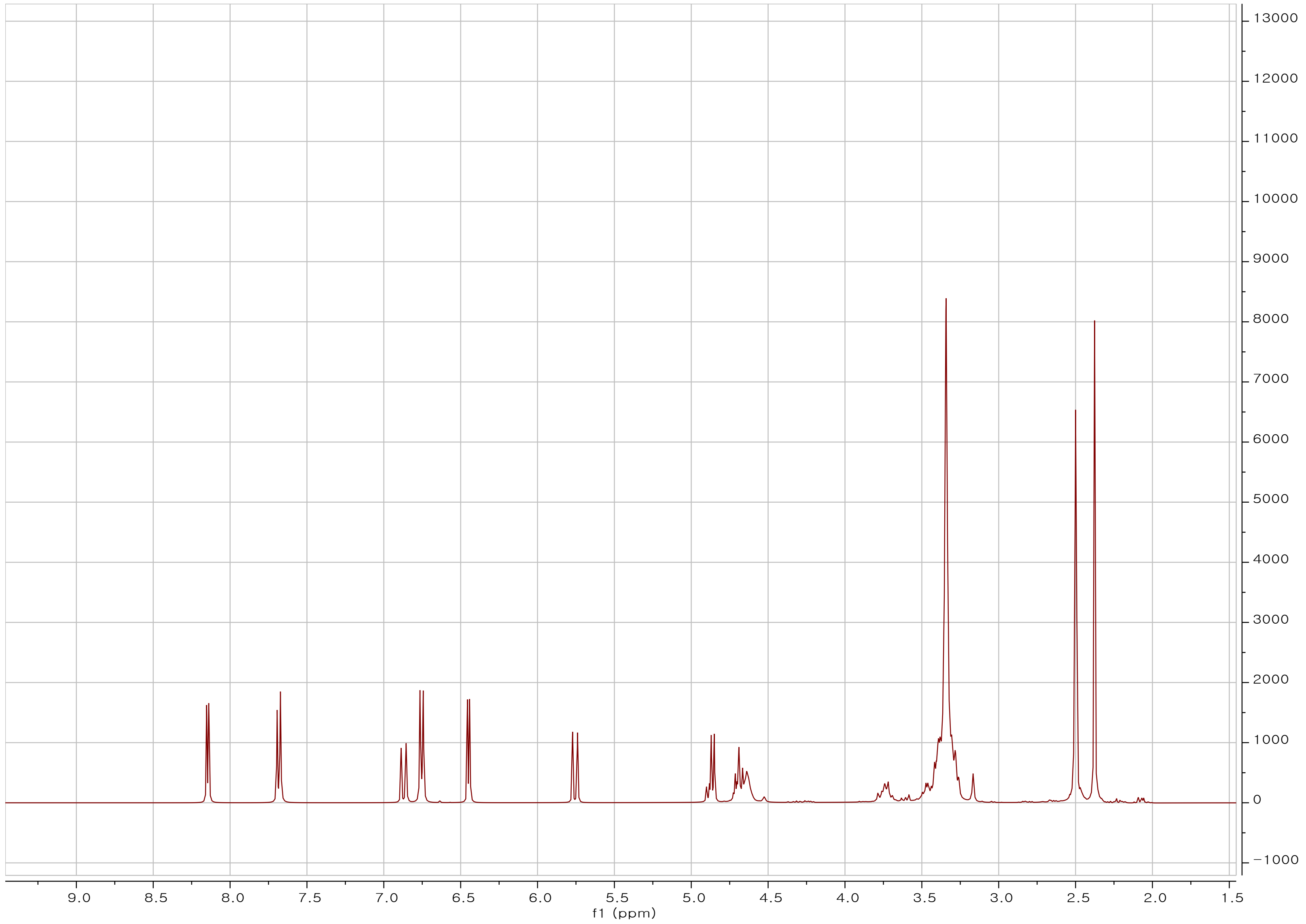

S2. The ^{13}C NMR spectrum of compound **3**

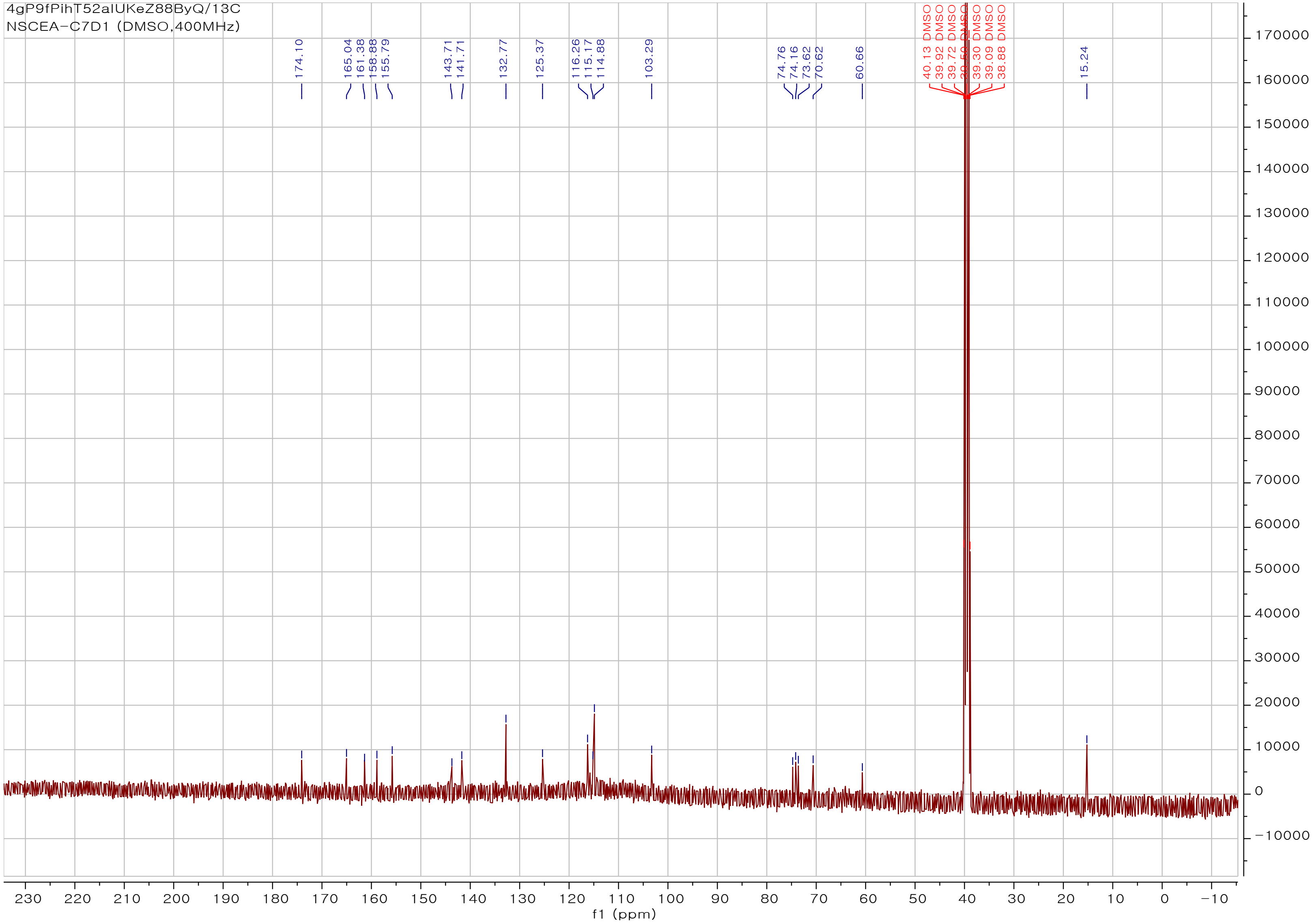

S3. The ^{1}H-^{1}H COSY spectrum of compound **3**

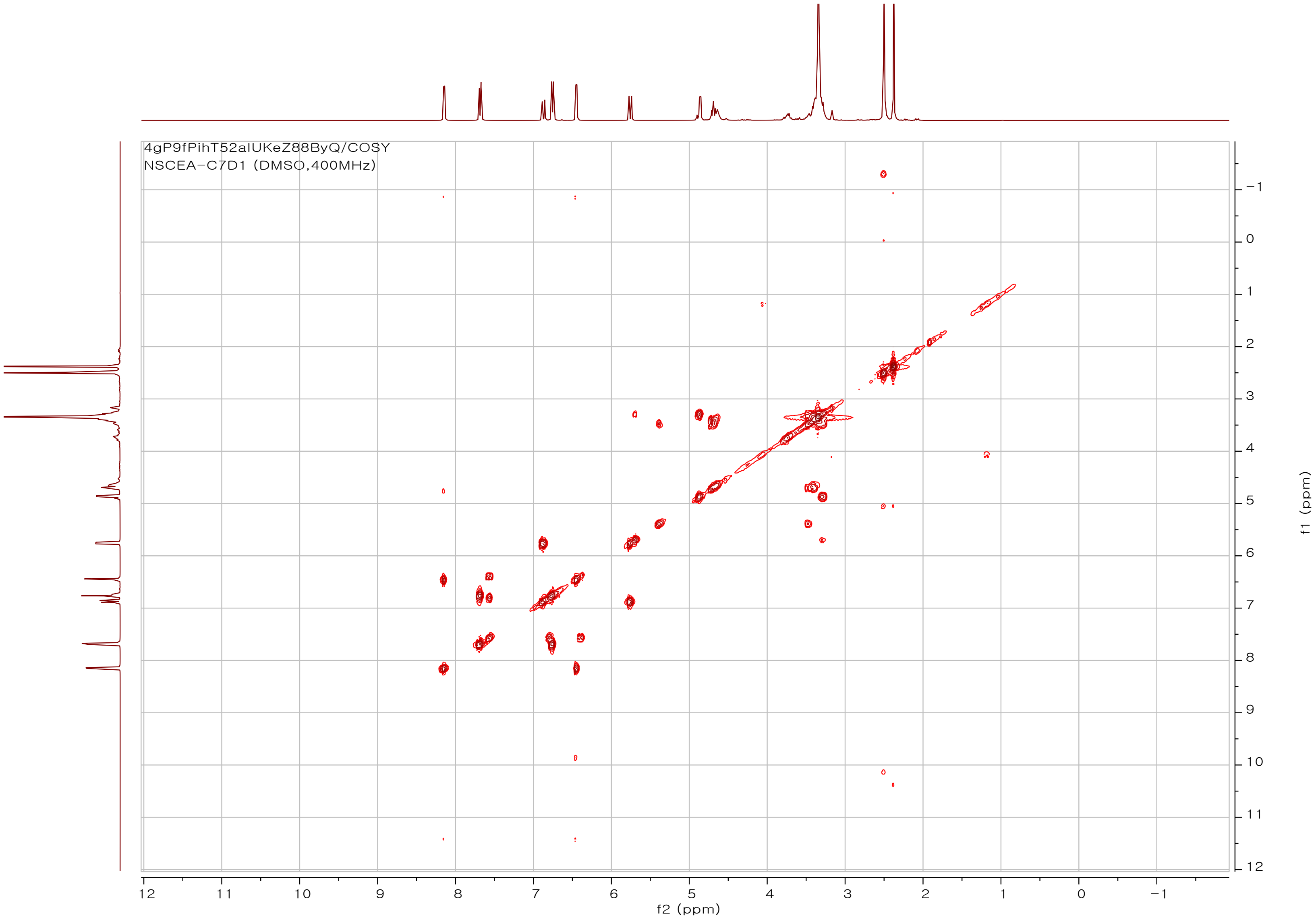

S4. The ^{1}H-^{1}H NOESY spectrum of compound **3**

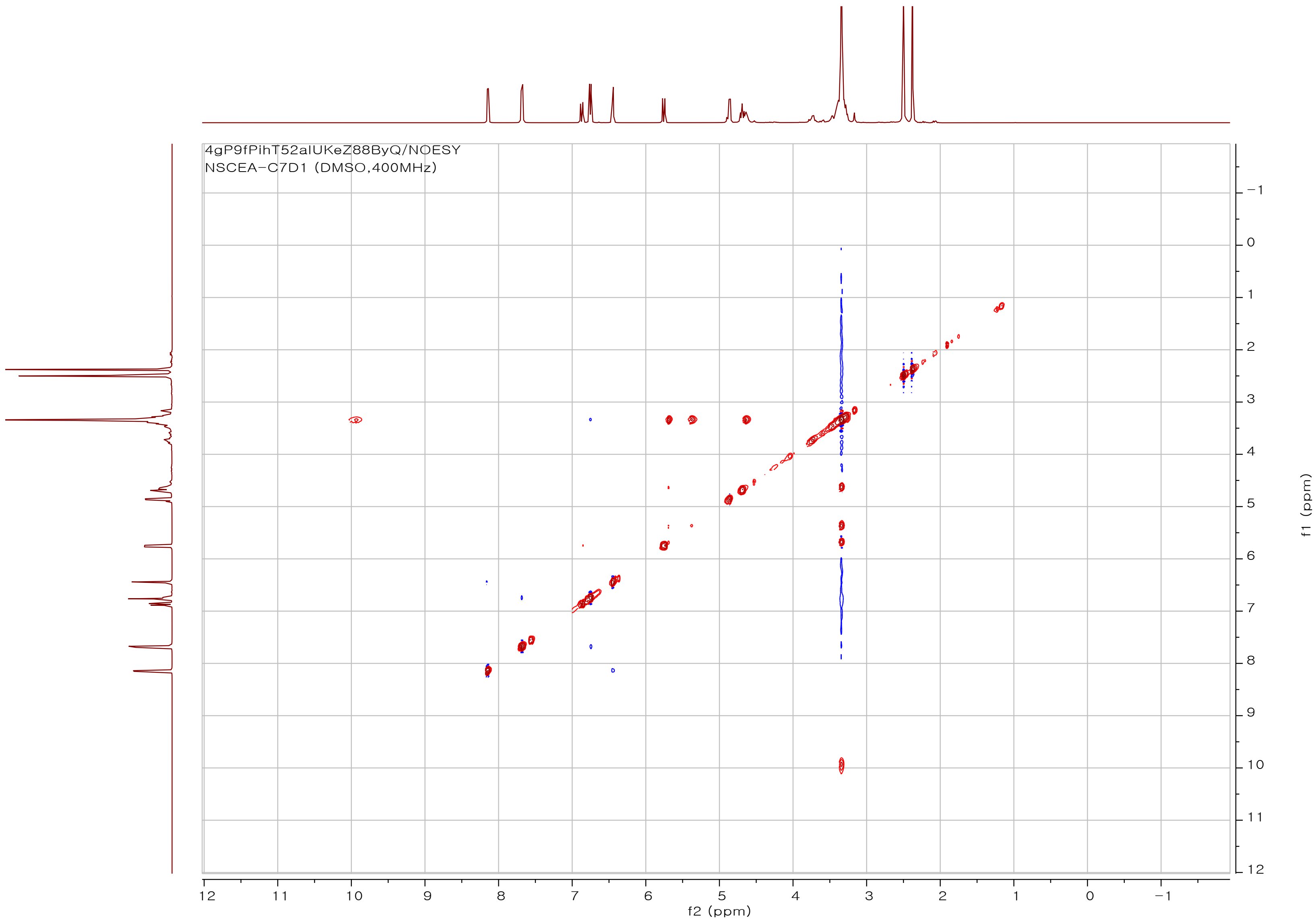

S5. The HMQC spectrum of compound **3**

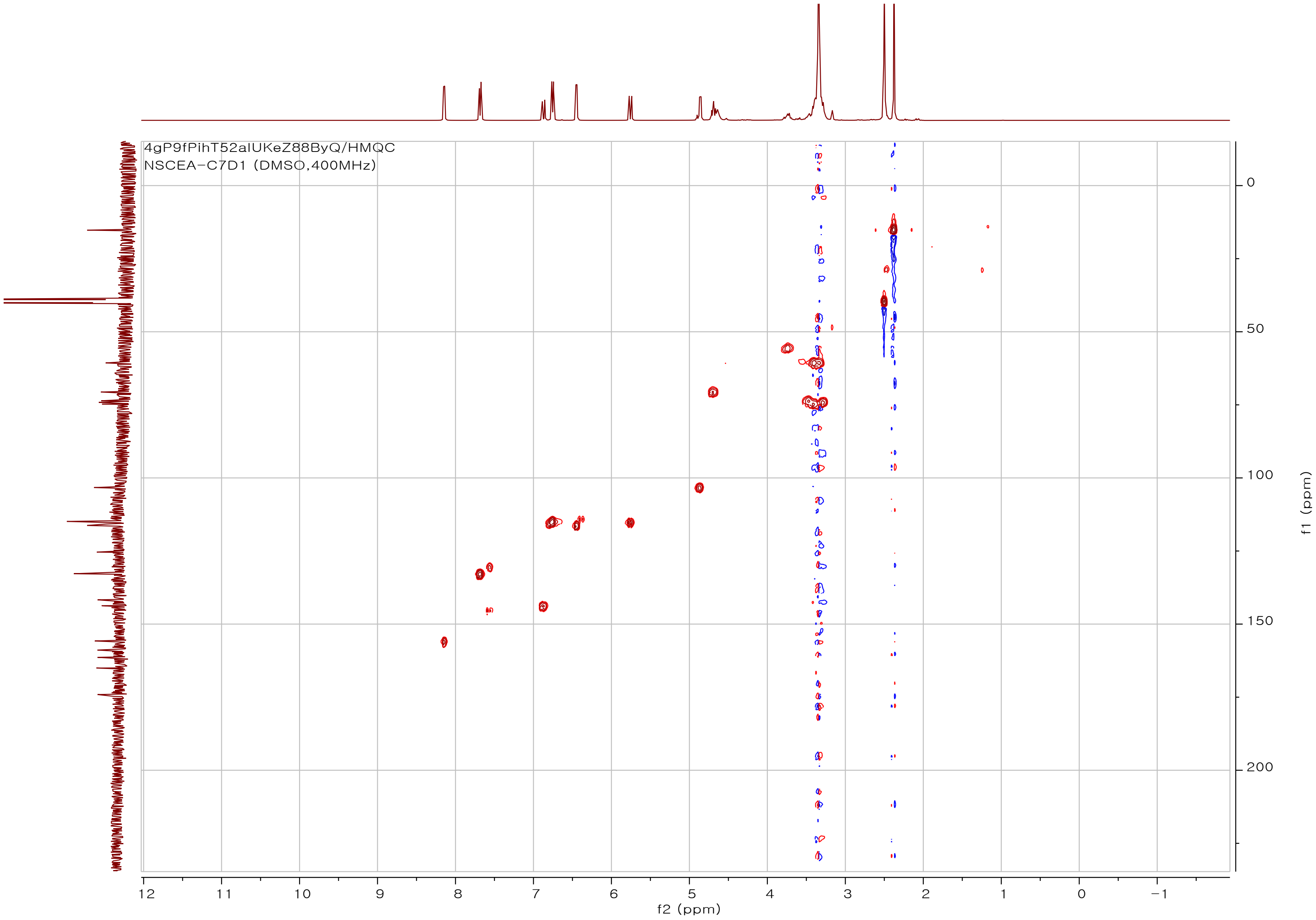

S6. The HMBC spectrum of compound **3**

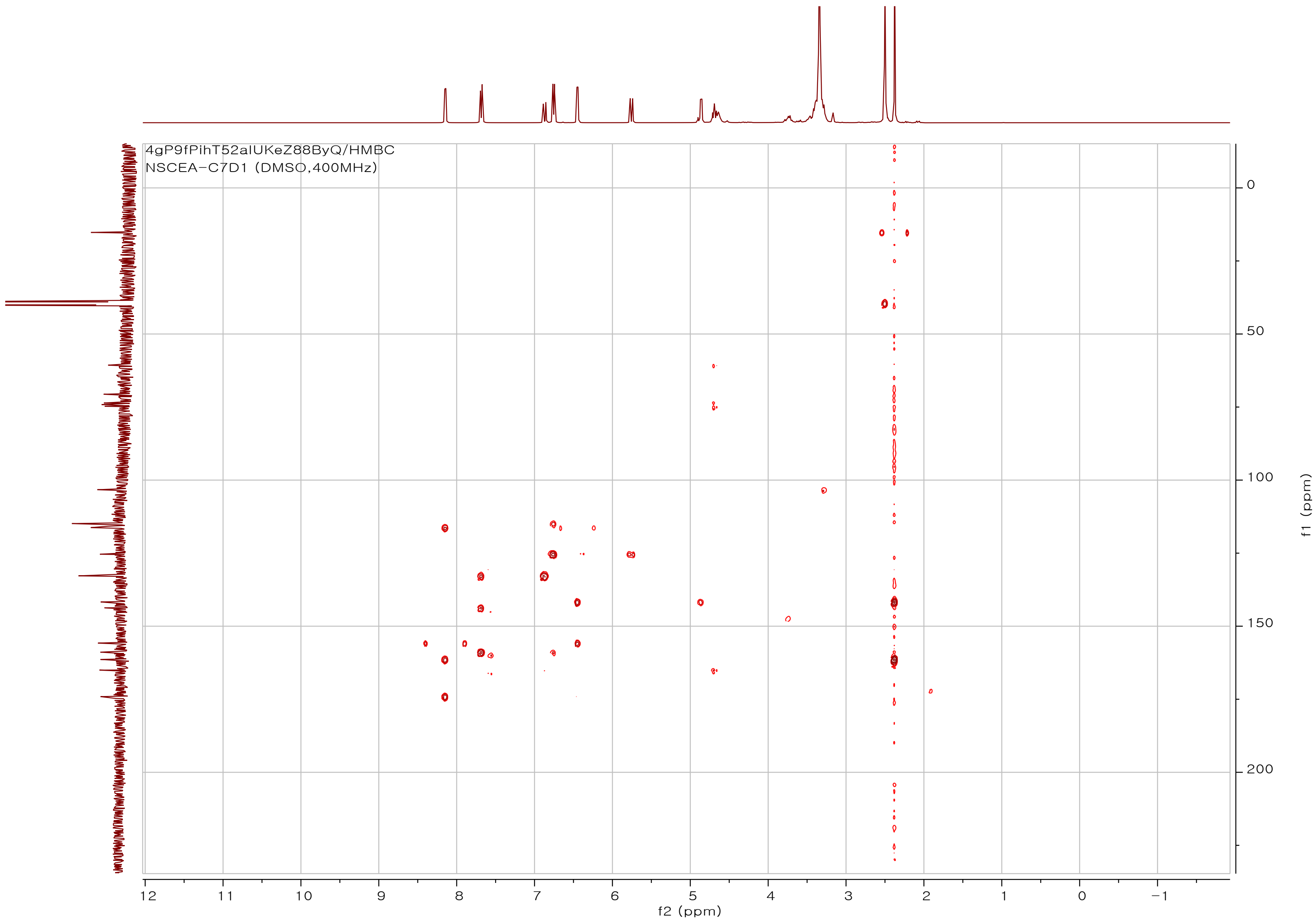

Supplement: RA-009-C8RA09743G-s001 [file RA-009-C8RA09743G-s001.pdf]
